# Dynamic changes in the migratory microbial components of colon tissue during different periods of sepsis in an LPS-induced rat model

**DOI:** 10.3389/fcimb.2023.1330087

**Published:** 2024-01-15

**Authors:** Hao Xu, Jia You, Wenqin He, Lingpeng Pei, Yue Han, Xueer Wang, Zhigang Tian, Xiwei Zheng, Enqi Wu, Yaqin Ling

**Affiliations:** ^1^ School of Pharmacy, Minzu University of China, Beijing, China; ^2^ Key Laboratory of Ethnomedicine (Minzu University of China), Ministry of Education, Beijing, China; ^3^ Department of Obstetrics and Gynecology, The Seventh Medical Center of Chinese PLA General Hospital, Beijing, China; ^4^ Department of Respiratory and Critical Care Medicine, General Hospital of Ningxia Medical University, Yinchuan, China

**Keywords:** sepsis, microbiota, microbial translocation, LPS, colon tissue

## Abstract

Previous studies have shown that bacterial translocation may play an important role in worsening gastrointestinal injury during sepsis. However, the dynamics of specific microbiota components in intestinal tissues at different sepsis stages remain unclear. Rats receiving intraperitoneal lipopolysaccharide (LPS) were sacrificed at 12 h and 48 h post-injection. Routine blood, serum cytokines, and microbiota in colon tissue, colonic contents, and lung tissue at different time points were assessed. Migratory microbial components in colonic tissue at 12 h and 48 h post-LPS were identified using source tracking, characteristic component identification, and abundance difference analyses. Colonic tissue microbiota changed dynamically over time after LPS injection, involving translocation of microbial components from colon contents and lung tissue at different time points. Bacteria migrating to colon tissue at 12 h sepsis were mainly from colonic contents, while those at 48 h were predominantly from the lung tissue. The migratory microbial components in colon tissue were widely associated with blood indicators and colonizing genus abundance and microbiota functionality in colon tissue. In this study, the temporal dynamics of bacterial translocation from various sources into colon tissues at different sepsis progression stages were characterized for the first time, and the species composition of these migrating microbes was delineated. These bacterial migrants may contribute to the pathophysiological processes in sepsis through direct interactions or indirectly by modulating colonic microbiota community structure and function.

## Introduction

Sepsis, a condition in which the body responds improperly to infection, can result in life-threatening organ dysfunction (Singer et al., 2016). The United States Centers for Disease Control and Prevention (CDC) estimates that sepsis claims the lives of 270,000 of the 1.7 million adults in the US who develop the condition each year (Buchman et al., 2020). Li et al. estimated that 4.8–6.1 million hospitalized cases of sepsis occurred annually in China from 2017 to 2019 (Weng et al., 2023). With the aging of the population, clinical invasive surgery, mechanical ventilation, and other technologies are widely used, resulting in an increase in the incidence and mortality of severe sepsis year by year. Up to 45% of patients with septic shock will suffer from multiple organ dysfunction, and the mortality rate is close to 50% (Cuthbertson et al., 2013; Singer et al., 2016), making the treatment of sepsis patients a colossal challenge for both clinicians and researchers.

The gastrointestinal tract is the most easily and frequently damaged organ during sepsis (Zheng and Zhu, 2016; Lelubre and Vincent, 2018; Otani et al., 2020). The inflammatory response caused by bacterial translocation may be an important factor in the aggravation of gastrointestinal injury (Deitch, 2012; Assimakopoulos et al., 2018). On the one hand, the increase in intestinal permeability caused by various factors can lead to the translocation of pathogenic bacteria from the intestinal contents to the intestinal tissue. This, in turn, triggers the recruitment of inflammatory cells and the release of pro-inflammatory mediators, culminating in apoptosis of intestinal cells and worsening tissue injury (Bianchi, 2007; Hunninghake et al., 2010; Oliveira-Nascimento et al., 2012; Haussner et al., 2019). On the other hand, the entrance of pathogenic bacteria and their products in the blood system through intestinal lymphatic vessels and mesenteric lymph nodes could trigger systemic inflammatory reactions (Assimakopoulos et al., 2018; Ma et al., 2021). The release of a variety of inflammatory mediators can cause damage to the vascular endothelium and result in the formation of microthrombi in intestinal tissues, leading to intestinal mucosal erosion, bleeding, cell necrosis, and detachment, which further increases intestinal permeability, forming a vicious cycle (Chelazzi et al., 2015; Ince et al., 2016).

However, not much is known so far about the dynamics of specific components of the microbiota in intestinal tissues at different stages of sepsis. Intraperitoneal injection of Lipopolysaccharides (LPS) can rapidly induce systemic inflammatory responses commonly seen in early sepsis, such as fever, cytokine storm, and multiple organ dysfunction. Due to its simple operation and good reproducibility, it is commonly used to study the pathophysiological changes in sepsis (Korneev, 2019). In this study, we established a sepsis rat model by intraperitoneal injection of LPS. Considering that both the lungs and colon can directly communicate with the external environment and harbor their own resident microbiota, we analyzed the microbiota in colon tissue, colon content, and lung tissue at 12 and 48 hours after LPS injection to explore the dynamic changes in microbial components of colon tissue and the origin of migrating microbes during the development of sepsis.

## Materials and methods

### Experimental animals and ethical statement

Eighteen male, 8-week-old, specific pathogen-free Sprague-Dawley rats weighing 200 ± 20 g were obtained from SPF Biotechnology Co., Ltd. (Beijing, China). Rats were housed in standard polypropylene shoebox cages (42×20.5×20 cm) on hardwood chip bedding in a designated room with a 12/12-h light/dark cycle at 24–26°C and a relative humidity of 50%. They had free access to water and were fed a standard diet. All animal experiments were performed in accordance with the Animal Research: Reporting of Experiments in Vivo (ARRIVE) guidelines. The study protocol was approved by the Ethics Committee of the Minzu University of China (Beijing, China; No. ECMUC2023005AO).

### Establishment of the sepsis model

The experiment was conducted with rats housed in a Specific Pathogen Free (SPF) level facility. Prior to grouping and treatment, both the model and control rats went through a one-week acclimatization period under identical conditions. Eighteen male Sprague-Dawley rats were randomly divided into three groups (n=6 per group) using a random number generator to ensure unbiased selection. Rats in the LPS12 and LPS48 groups received an intraperitoneal injection of LPS (Escherichia coli O55: B5; Cat. No. L2880, Sigma-Aldrich, St. Louis, MO, USA; 10 mg/kg body weight), dissolved in 0.9% NaCl solution, following the dosing strategy based on the protocol established in the study by Li T et al. (Li et al., 2013). The rats in these groups were sacrificed after 12 and 48 h, respectively. Rats in the control group received an intraperitoneal injection of 0.9% NaCl (Cat. No. G4702, Servicebio, Wuhan, China) solution and were sacrificed after 48 h.

### Sample collection

For sample collection, all rats were anesthetized with isoflurane and blood was collected from the abdominal aorta. Whole blood was used for routine blood tests. Serum was collected by centrifuging the whole blood at 1000 g for 10 min and then stored at −80°C prior to cytokine assays. The colon was cut at 8–11 cm from the anus using a sterile instrument. Approximately 0.5 g of colon content was collected, and colon tissue sections (~1 cm) were cut, then washed three times with PBS (Cat. No. G4202, Servicebio, Wuhan, China). Subsequently, both the colon content and colon tissue sections were separately placed in sterile tubes containing a 4 M guanidine thiocyanate solution (Cat. No. 50983, Sigma-Aldrich, St. Louis, MO, USA) for microbiota analysis. The right middle lung lobe was harvested immediately by opening the chest with sterile scissors and placed in sterile tubes containing 4 M guanidine thiocyanate solution for microbiota analysis. All samples for microbiota analysis were stored at −80°C prior to DNA extraction.

### Routine blood and serum cytokines testing

A total of 24 clinical hematological parameters were determined using an Automated Hematology Analyzer (Sysmex, Tokyo, Japan). The serum levels of four cytokines, including interleukin-1β (IL-1β), IL-6, IL-10, and tumor necrosis factor-α (TNF-α), were quantified using enzyme-linked immunosorbent assay (ELISA) kits (Cat. No. ml037361; Cat. No. ml064292; Cat. No. ml037371; Cat. No. ml002859; Shanghai Enzyme-linked Biotechnology Co., Ltd., Shanghai, China). The assay was conducted on 6 biological replicates per treatment group, with each measurement repeated 3 times (technical replicates).

### Microbiota sequencing

Total bacterial DNA was extracted using a Power Soil DNA Isolation Kit (MO BIO Laboratories, Inc., Carlsbad, CA, USA). Quality of the DNA samples was checked by calculating the ratios of absorbance at 260/280 nm and 260/230 nm. The V3–V4 hypervariable region of the bacterial 16S rRNA gene was amplified using the primers 338F (5′-ACTCCTACGGGAGGCAGCAG-3′) and 806R (5′-GGACTACHVGGGTWTCTAAT-3′) combined with adapter and barcode sequences. Second-generation sequencing of purified pooled PCR products was performed on a HiSeq 2500 platform (Illumina, Inc., San Diego, CA, USA; 2×250 paired ends) at Biomarker Technologies Corporation (Beijing, China). The assay was conducted on 6 biological replicates per treatment group.

### Bioinformatic analyses

Raw sequences were processed using the UNOISE pipeline of the Usearch v11.0.667linux64 program (www.drive5.com/usearch/). The high-quality sequences were classified as zero radius operational taxonomic units (ZOTU) and the classification information of each ZOTU sequence was annotated using the Ribosomal Database Project (RDP) classifier with a confidence threshold of 80%. The species-level taxonomic annotation of ZOTUs was accomplished by further aligning their unique sequences to the Ezbiocloud database (www.ezbiocloud.net). The ZOTU was randomly sampled (42,030 reads, the minimum number of reads in a sample) to obtain equal sequencing depth between samples. The Shannon, PD_whole_tree, and Observed_otus indices were calculated using the QIIME 1.91 pipeline. The Jaccard and Bray–Curtis distance matrices were calculated from the ZOTU table, and principal coordinate analysis (PCoA), distance-based redundancy analysis (db-RDA), and adonis tests were performed using the “vegan” package in R. Source tracking of the microbiota of colon tissue was conducted using the fast expectation-maximization microbial source tracking (“FEAST”) R package (Shenhav et al., 2019). To identify the characteristic microbial components of each sample group, the characteristic ZOTUs that were significantly distinct from other groups were predicted for each microbial community group based on the ZOTU abundance data using the “interspecies” package in R. The KEGG-based functional pathway profiles of the microbial communities were predicted using the “Tax4Fun” R package, based on ZOTU abundance data with taxonomy assigned using the SILVA Reference database (release 123) (Aßhauer et al., 2015). The “psych” R package was used to calculate correlation coefficients between variables while performing significance tests.

### Statistical analyses

R software (version 3.52; R Foundation for Statistical Computing, Vienna, Austria) was used to perform all statistical analyses. A Shapiro–Wilk test was used to test for a normal distribution, and analysis of variance (ANOVA) or the Kruskal–Wallis test was used to evaluate differences in measured variables among the groups. Spearman’s ρ correlation analysis was performed to evaluate the associations between different variables. p-values were corrected for multiple tests using the false discovery rate (FDR) method.

## Results

### Dynamic changes in blood routine and serum cytokine indices at different time points after LPS injection

**Figure 1:**
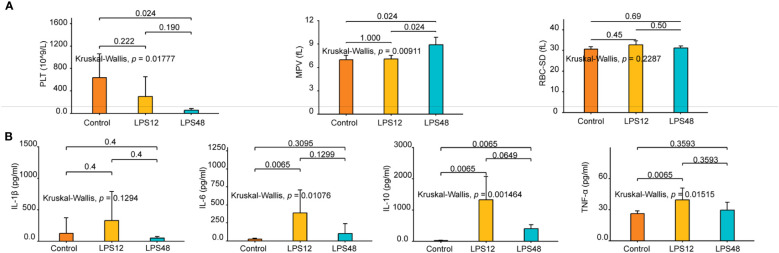
Changes in hematological parameters and serum cytokine levels at indicated time points after LPS injection. **(A)** Of the 24 hematological parameters examined, three, namely platelet count (PLT), mean platelet volume (MPV), and red blood cell distribution width standard deviation (RBC-SD), showed different degrees of variation between different treatment groups. **(B)** The serum levels of four cytokines—IL-1β, IL-6, IL-10, and TNF-α—varied among different treatment groups. Data shown represent the mean ± standard deviation of 6 biological replicates per group.

In the routine blood testing results, three indicators, namely platelet count (PLT), mean platelet volume (MPV), and red blood cell distribution width standard deviation (RBC-SD), showed different degrees of variation between different treatment groups (**Figure 1A**). Among them, PLT gradually decreased in the LPS12 and LPS48 groups compared with the control group, and there was a significant difference between the LPS48 group and the control group (*P* < 0.05). MPV was similar between the control group and the LPS12 group, while it significantly increased in the LPS48 group (*P* = 0.024). RBC-SD was higher in the LPS12 group than in the control group, and it was lower in the LPS48 group than in the LPS12 group, but these differences were not statistically significant.

With respect to serum cytokines, dynamic changes in the levels of IL-1β, IL-6, IL-10, and TNF-α were observed over time following LPS administration. At 12h after LPS stimulation, the concentrations of all four cytokines were elevated to varying extents compared to the control group. However, at the 48h time point, the levels of the cytokines showed declining trends compared to the 12h data. Further statistical analyses shows that the changes in IL-6, IL-10 and TNF-α levels across the three experimental groups were statistically significant, whereas differences in IL-1β concentrations were not significant between groups. Specifically, serum IL-6 and TNF-α concentrations were markedly higher in the LPS12 group compared to the control group. Serum IL-10 levels were significantly increased in both LPS12 and LPS48 groups relative to the control (**Figure 1B**).

### No significant change in α-diversity of colon tissue microbiota at different time points after LPS injection

We performed high-throughput sequencing analysis of the microbiomes of colon tissue, lung tissue, and colon content of 18 rats. A total of 3,063,842 clean reads were collected after trimming and filtering. A ZOTU table with 20,238 ZOTUs was ultimately generated and further used for data analysis. A total of 19,945 ZOTUs were successfully annotated by the RDP classifier, comprising 30 phyla, 67 classes, 123 orders, 224 families, and 443 genera.

**Figure 2:**
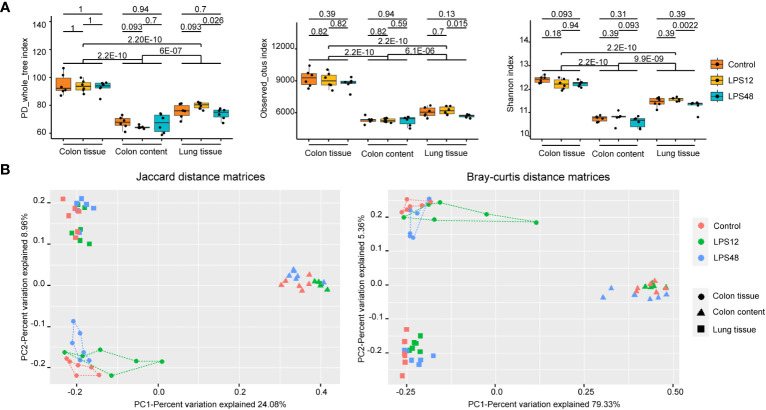
Alpha- and beta-diversity analysis of the microbiota of three sampling sites in different treatment groups. **(A)** The PD_whole_tree, Observed_otus, and Shannon α-diversity indices were significantly different among the three sampling sites. Data shown represent the mean ± standard deviation of 6 biological replicates per group. **(B)** The PCoA diagrams of the Jaccard and Bray–Curtis distance matrices show that the samples of colon tissue, lung tissue, and colon content were all gathered in distinct positions. The db-RDA analysis (*P* = 0.001 and adjusted *r* ^2 =^ 0.301 for the Jaccard distance matrix; *P* = 0.001 and adjusted *r* ^2 =^ 0.366 for the Bray–Curtis distance matrix) and adonis analysis (*P* = 0.001 and *r* ^2 =^ 0.327 for the Jaccard distance matrix; *P* = 0.001 and *r* ^2 =^ 0.712 for the Bray–Curtis distance matrix) both confirmed the significant association between the different sample sites and bacterial community structures. Each point represents an individual sample, with 6 biological replicates included for each treatment group.

Three α-diversity indices (PD_whole_tree, Observed_otus, and the Shannon index) were calculated for each of the samples. As expected, among the different sites, all three α-diversity indices differed significantly between colon tissue, lung tissue, and colon content, with the highest α-diversity in colon tissue, followed by lung tissue and then colon content. Within the same site, although indices of PD_whole_tree, Observed_otus and Shannon index exhibited a significantly decreased trend in the lung tissue microbiota of the LPS48h group, no significant differences were observed across these α-diversity indices between the different LPS injection time point groups for colon tissue and colon content samples (**Figure 2A**).

### Colon tissue microbiota structure undergoes dynamic changes at different time points of LPS injection

To explore the changes of microbiota structure, the Jaccard distance matrix based on the presence or absence of data and the Bray–Curtis distance matrix based on the abundance data were calculated from the ZOTU tables of all samples. In the PCoA diagrams of both sets of distance matrices, samples of colon tissue, lung tissue, and colon content were all gathered in distinct positions (**Figure 2B**). The db-RDA analysis and adonis analysis both confirmed the significant association between the different sample sites and bacterial community structures (*P* = 0.001). It could be seen that the distance between colon tissue samples (dotted) and lung tissue samples (squares) is shorter than the distance between colon tissue samples and colon content (triangles) samples from both PCoA diagrams, indicating that the microbiota from colon tissue and lung tissue share a higher similarity than microbiota from colon content.

When further investigating the distribution of samples from different treatment groups at each sampling site, the trend of separation occurring in different treatment groups can also be seen. It is noteworthy that in the colon tissue samples (dotted), the samples in the LPS12 group (green line) were closer to the area of the colon content samples (triangles) and the samples in the LPS48 group (blue line) were closer to the lung tissue samples (squares), suggesting that the colon tissue in the LPS12 group may contain some bacteria that migrated from the colon content, while the colon tissue in the LPS48 group may contain some bacteria that migrated from the lung tissue.

### Early colon tissue migrant microbes mainly derived from colon contents while late ones mainly derived from lung tissue in LPS-induced sepsis

**Figure 3:**
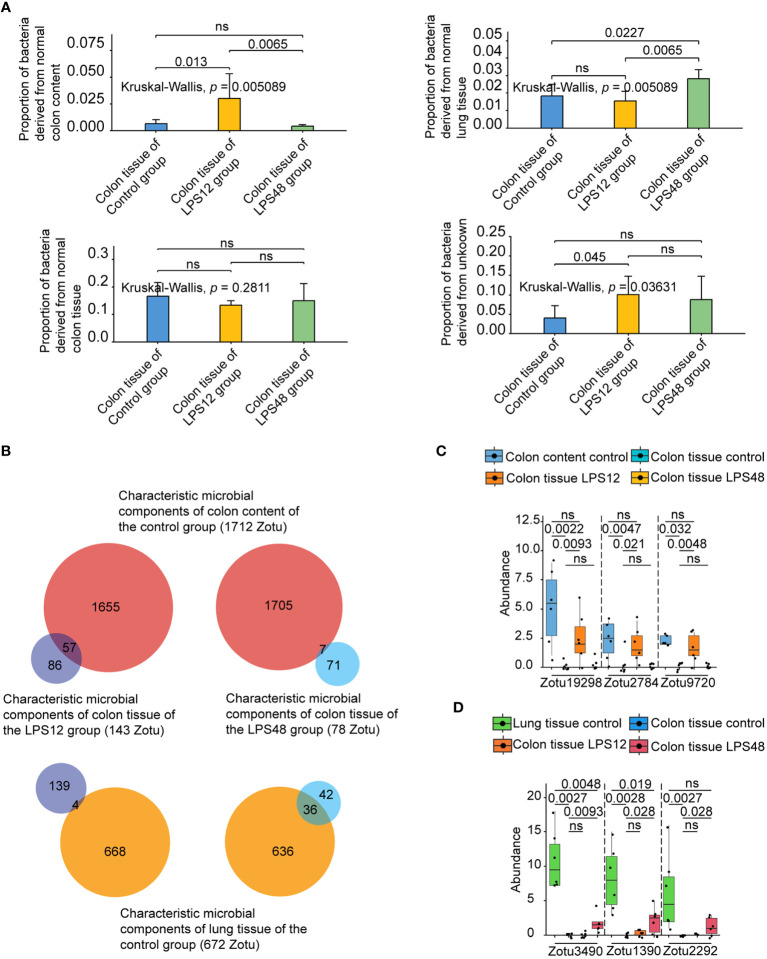
Source tracking analysis and abundance comparison of the characteristic microbial components of colon tissue at different time points after LPS injection. **(A)** Source tracking analysis of microbiota of colon tissue at different time points after LPS injection. **(B)** Intersection analysis of the characteristic microbial components of colon content and lung tissue of the control group with the characteristic microbial components of colon tissues of the LPS12 and LPS48 groups. **(C)** Comparison of the abundance (copy number) of representative colon content source characteristic ZOTUs in the control colon content, control colon tissue, and LPS12 colon tissue. **(D)** Comparison of the abundance of representative lung tissue source characteristic ZOTUs in the control lung tissue, control colon tissue, and LPS12 colon tissue. Data shown represent the mean ± standard deviation of 6 biological replicates per group.

To determine whether bacteria originating from colon content and lung tissue were present in colon tissue at different time points after LPS injection, we performed source tracking analysis on the colon tissue microbiome of the LPS12 and LPS48 groups, using the microbiome of colon tissue, lung tissue, and colon content of the control group as the normal reference sources of different sites in the input to FEAST. The results showed that the proportion of bacteria in colon tissue derived from normal colon contents (bacteria in colon content of control group) was significantly higher in the LPS12 group than in the LPS48 group, while the proportion of bacteria in colon tissue derived from normal lung tissue (bacteria in lung tissue of control group) was significantly higher in the LPS48 group than in the LPS12 group. The proportions of bacteria derived from normal colon tissue (bacteria in colon tissue of control group) and unknown sources were not significantly different between the two groups (**Figure 3A**).

To further identify the specific microbial components (ZOTU) migrating from other sites to colon tissue at different time points following LPS injection, we first compared the microbial components in colon tissue, lung tissue, and colon content of the control group with the interspecies package in R. It was found that 1,712 of the 12,443 ZOTUs in the colon content of the control group were characteristic microbial components that were different from the other two habitats, and 672 of the 13,898 ZOTUs in the lung tissue of the control group were characteristic microbial components that were different from the other two habitats (a detailed list of those ZOTUs and their exact sequences are provided in the **Supplementary Table 1**). Then, by comparing the colon tissue microbiota of the control, LPS12, and LPS48 groups, we found that 143 of the 17,220 ZOTUs in the colon tissue of the LPS12 group were characteristic microbial components different from the other two habitats, and 78 of the 16,115 ZOTUs in the colon tissue of the LPS48 group were characteristic microbial components different from the other two habitats. Finally, by performing intersection analysis of the characteristic microbial components of colon content and lung tissue of the control group (1712 and 672 ZOTUs, respectively)with the characteristic microbial components of colon tissues of the LPS12 and LPS48 groups(143 and 78 ZOTUs, respectively), we found that 57 ZOTUs in the characteristic microbial components of colon tissue in the LPS12 group were the characteristic microbial components of colon content in the control group (1712 ZOTUs), and 4 ZOTUs were the characteristic microbial components of lung tissue in the control group (672 ZOTUs) (**Figure 3B**). The chi-square test revealed a statistically significant difference in the proportions between these two groups (**Supplementary Data Sheet 1**). Among the characteristic microbial components of the colon tissue in the LPS48 group, 36 ZOTUs were characteristic microbial components of lung tissue in the control group (672 ZOTUs), and 7 ZOTUs were characteristic microbial components of the colon content in the control group (1712 ZOTUs). The chi-square test revealed a statistically significant difference in the proportions between these two groups (**Supplementary Data Sheet 1**). These data further suggest that the migrating microbial components in the colon tissue at 12 h after LPS injection were mainly derived from the colon contents, whereas the migrating microbial components in the colon tissue at 48 h after LPS injection were mainly derived from lung tissue.

In the comparison of the abundance (copy number) of the abovementioned intersection characteristic ZOTUs in different sites, it was confirmed that all 57 potential colon content source characteristic ZOTUs had high abundance in colon content in the control group, very low abundance in colon tissue in the control group, and significantly increased abundance in colon tissue in the LPS12 group (**Figure 3C**; **Supplementary Data Sheet 2**). Similarly, the 36 potential lung tissue source characteristic ZOTUs also conformed to the pattern of high abundance in the lung tissue of the control group, very low abundance in the colon tissue of the control group, and a significant increase in the abundance in the colon tissue of the LPS48 group (**Figure 3D**; **Supplementary Data Sheet 2**). These data further verified that the 57 characteristic ZOTUs of colon tissue in the LPS12 group were derived from colon content and 36 characteristic ZOTUs of colon tissue in the LPS48 group were derived from lung tissue.

### Species annotation of temporal migrating microbial components

We uploaded the sequences of abovementioned characteristic ZOTUs derived from colon content and lung tissue to the EzBioCloud database for species annotation. The results showed that the 57 characteristic ZOTUs derived from colon contents were annotated to 26 species. Among those species, *Roseburia cecicola* and *Oscillibacter PAC001185_s* were the predominant species, accounting for 8 and 6 ZOTUs, respectively. For the characteristic microbial components derived from lung tissue, 36 characteristic ZOTUs were annotated to 19 species. Among them, *Rouxiella silvae*, *Pantoea hericii*, and *Bifidobacterium panos* were the predominant species, accounting for 8, 6, and 5 ZOTUs, respectively. Detailed information on species-specific annotations is shown in **Table 1**.

**Table 1:** Summary of species annotation of the characteristic ZOTUs of different sources.

Source from colon content			Source from lung tissue		
Species annotation	Number of Zotu	Zotu	Species annotation	Number of Zotu	Zotu
*Roseburia cecicola*	8	Zotu10181; Zotu4620; Zotu4540; Zotu10624; Zotu8100; Zotu9720; Zotu6413; Zotu19792	*Rouxiella silvae*	8	Zotu5138; Zotu3490; Zotu2876; Zotu2842; Zotu2328; Zotu1590; Zotu1461; Zotu1390
*Oscillibacter PAC001185_s*	6	Zotu2784; Zotu5266; Zotu6024; Zotu7664; Zotu3270; Zotu2880	*Pantoea hericii*	6	Zotu822; Zotu4701; Zotu4180; Zotu3292; Zotu2963; Zotu2439
*RAYR_g PAC001082_s*	3	Zotu465; Zotu408; Zotu407	*Bifidobacterium panos*	5	Zotu857; Zotu692; Zotu589; Zotu505; Zotu1388
*Agathobaculum AY239438_s*	3	Zotu3155; Zotu7996; Zotu2703	*Saccharimonas PAC001343_s*	2	Zotu3903; Zotu2292
*Prevotella hominis*	3	Zotu1231; Zotu838; Zotu782	*Rahnella variigena*	2	Zotu2233; Zotu1403
*Kineothrix alysoides*	3	Zotu3937; Zotu7496; Zotu8688	*Cetobacterium somerae*	1	Zotu2360
*Helicobacter rodentium*	3	Zotu56; Zotu81; Zotu23	*Chlamydia ibidis*	1	Zotu7194
*Pseudoflavonifractor PAC002490_s*	2	Zotu9935; Zotu7764	*Cloacibacterium normanense*	1	Zotu11703
*Sporobacter PAC001257_s*	2	Zotu15564; Zotu11711	*DQ395019_g JF344561_s*	1	Zotu12682
*Schaedlerella PAC001803_s*	2	Zotu13960; Zotu16786	*DQ811856_g DQ811856_s*	1	Zotu2989
*Ruminococcus EU137603_s*	2	Zotu2980; Zotu16282	*EU287221_g AB530216_s*	1	Zotu11036
*Lacrimispora saccharolytic*	2	Zotu1508; Zotu1443	*Fannyhessea vaginae*	1	Zotu2101
*Lachnotalea glycerini*	2	Zotu1918; Zotu2830	*Fulvivirga lutimaris*	1	Zotu19379
*Intestinimonas butyriciproducens*	2	Zotu14006; Zotu19298	*JF344531_g EU734944_s*	1	Zotu17283
*PAC001296_g AB626952_s*	2	Zotu11152; Zotu8194	*Olleya namhaensis*	1	Zotu7836
*Dehalobacterium AB755782_s*	2	Zotu4397; Zotu4829	*Pseudoxanthomonas sacheonensis*	1	Zotu4016
*Acetatifactor PAC001749_s*	1	Zotu7925	*Sandaracinus amylolyticus*	1	Zotu12790
*Alloprevotella Prevotellamassilia timonensis*	1	Zotu183	*Woeseia JN977186_s*	1	Zotu3775
*Anaerotignum AY239424_s*	1	Zotu5958			
*Angelakisella massiliensis*	1	Zotu17695			
*Coprococcus Lachnotalea glycerine*	1	Zotu2605			
*Enterocloster FCEY_s*	1	Zotu14092			
*PAC000672_g PAC000672_s*	1	Zotu14461			
*Oscillibacter FJ880774_s*	1	Zotu16309			
*Paramuribaculum FJ881296_s*	1	Zotu1215			
*Velocimicrobium porci*	1	Zotu11482			

### Migrating microbial components significantly associated with multiple blood indicators

**Figure 4:**
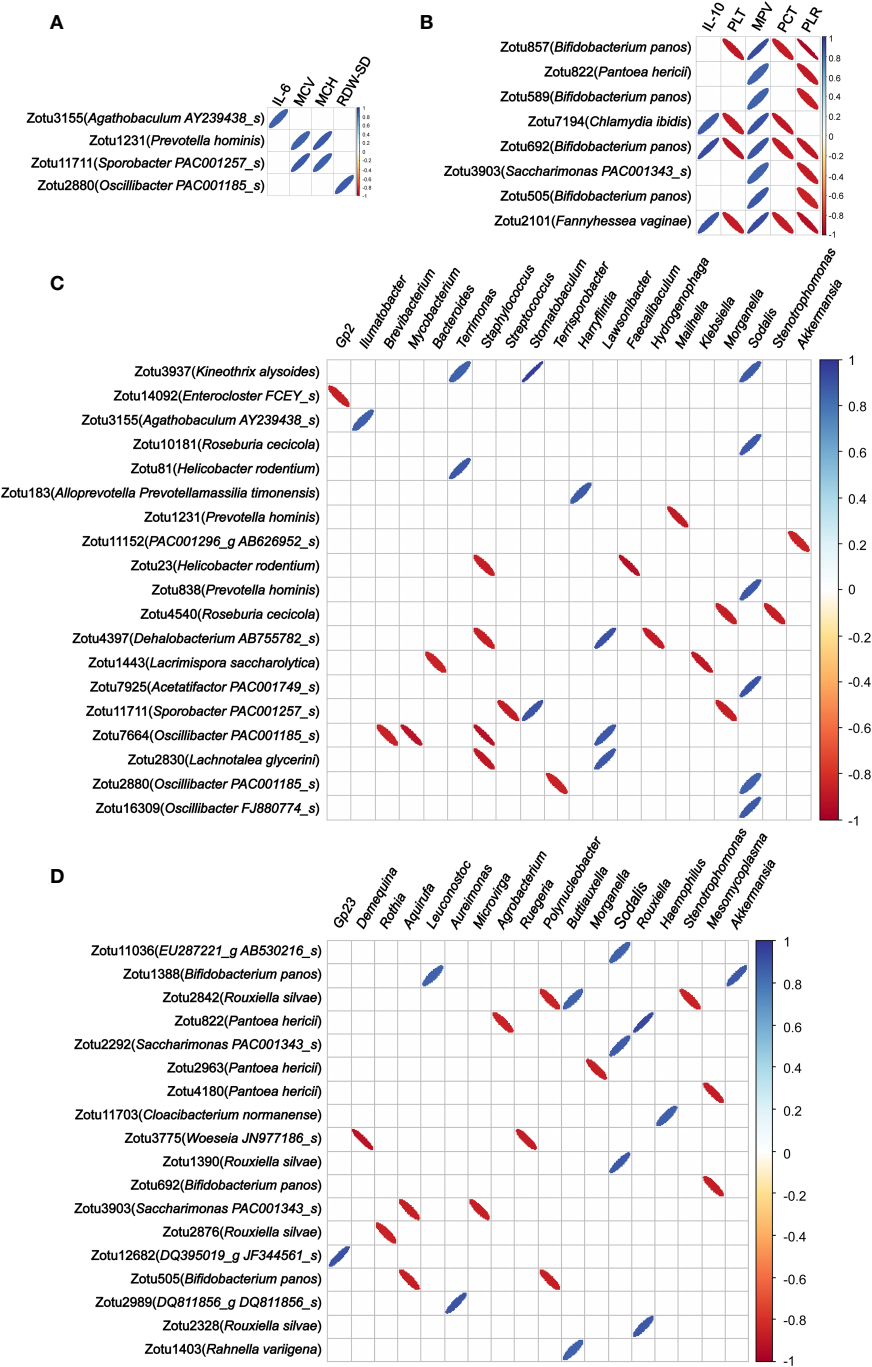
Correlations between the abundance of migrated microbial components in colon tissue and blood indicators and colonized genera of the colon tissue. **(A)** Correlation of the abundance of the microbial components in colon tissue migrating from colon content with blood indicators. **(B)** Correlation of the abundance of the microbial components in colon tissue migrating from lung tissue with blood indicators. **(C)** Correlation of the abundance of the microbial components in colon tissue migrating from colon content with colon tissue colonizing bacterial genera. **(D)** Correlation of the abundance of the microbial components in colon tissue migrating from lung tissue with colon tissue colonizing bacterial genera. Only statistically significant correlations with absolute values of the correlation coefficient greater than 0.85 were shown.

To explore associations between those migrating microbial components and blood indicators, we analyzed the correlation of the abundance of these microbial components in colon tissue migrating from colon content and lung tissue with routine blood parameters and serum cytokines. Among the 57 ZOTUs migrated from colon contents 12 h after LPS injection, we found that the abundance of 4 ZOTUs (belonging to *Agathobaculum AY239438_s*, *Oscillibacter PAC001185_s*, *Prevotella hominis*, and *Sporobacter PAC001257_s*, respectively) has 6 significant correlations with 4 blood indicators, including IL-6, mean corpuscular hemoglobin content (MCH), mean corpuscular volume (MCV), and RBC-SD (**Figure 4A**). Among the 36 ZOTUs migrated from lung tissue 48 h after LPS injection, we found that the abundance of 8 ZOTUs (4 of them belonging to *Bifidobacterium panos* and the remaining 4 belonging to *Pantoea hericii*, *Chlamydia ibidis*, *Sandaracinus amylolyticus*, and *Fannyhessea vaginae*, respectively) has 26 significant correlations with 5 blood indicators, including IL-10, PLT, platelet pressure volume (PCT), MPV, and large platelet ratio (P-LCR) (**Figure 4B**).

These results suggest that these migrating microbial components may be directly involved in the systemic pathophysiologic response of sepsis.

### Migrating microbial components significantly associated with colonizing genera, and microbiota functionalities of colon tissue

As regards the correlation of the abundance of these microbial components in colon tissue migrating from colon content and lung tissue with colon tissue colonizing bacterial genera, we found that the abundance of 19 out of 57 ZOTUs that migrated from colon contents 12 h after LPS injection has 33 significant correlations with 20 colon tissue colonizing bacterial genera (**Figure 4C**). Among the 36 ZOTUs that migrated from lung tissue 48 h after LPS injection, we found that the abundance of 18 ZOTUs has 25 significant correlations with 18 colon tissue colonizing bacterial genera (**Figure 4D**).

**Figure 5:**
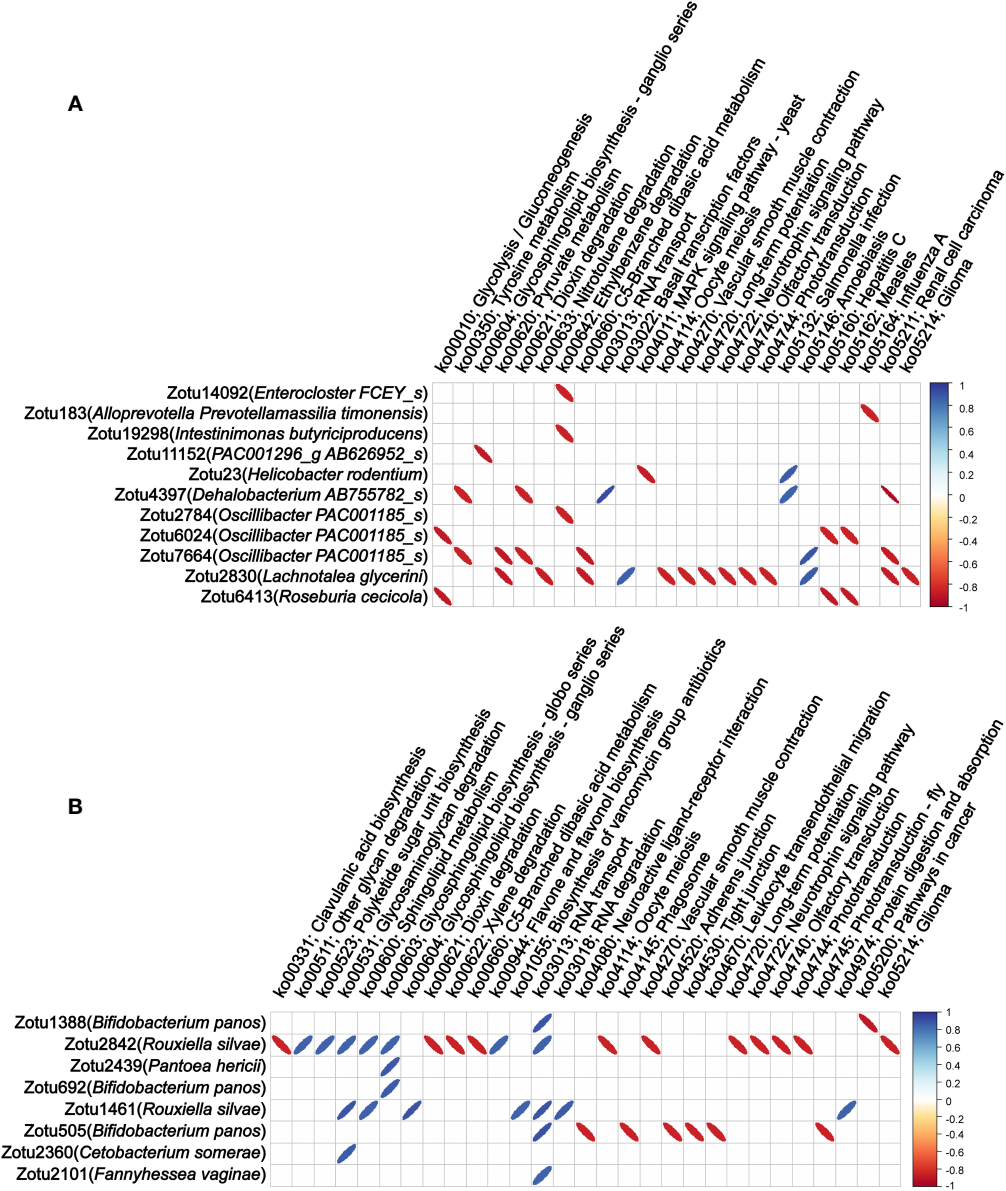
Correlations between the abundance of migrating microbial components and microbiota functionalities of colon tissue. **(A)** Correlation of the abundance of the microbial components in colon tissue migrating from colon content with microbiota functionalities of colon tissue. **(B)** Correlation of the abundance of the microbial components in colon tissue migrating from lung tissue with microbiota functionalities of colon tissue. Only statistically significant correlations with absolute values of the correlation coefficient greater than 0.85 were shown.

Finally, we analyzed the correlation of the abundance of these microbial components in colon tissue migrating from colon content and lung tissue with the microbiota functionalities of colon tissue. Among the 57 ZOTUs that migrated from colon contents 12 h after LPS injection, we found that the abundance of 11 ZOTUs has 37 significant correlations with 24 microbiota functionalities of colon tissue, and among 36 ZOTUs that migrated from lung tissue 48 h after LPS injection, we found that the abundance of 8 ZOTUs has 38 significant correlations with 29 microbiota functionalities of colon tissue. These findings suggest that bacteria migrating from other sites to colon tissue after LPS injection may have a widespread impact on the abundance of multiple bacterial genera colonizing the colon tissue and the microbiota functionalities of colon tissue. Detailed information of the correlations between the abundance of microbial components and microbiota functionalities is shown in **Figure 5**.

## Discussion

**Figure 6:**
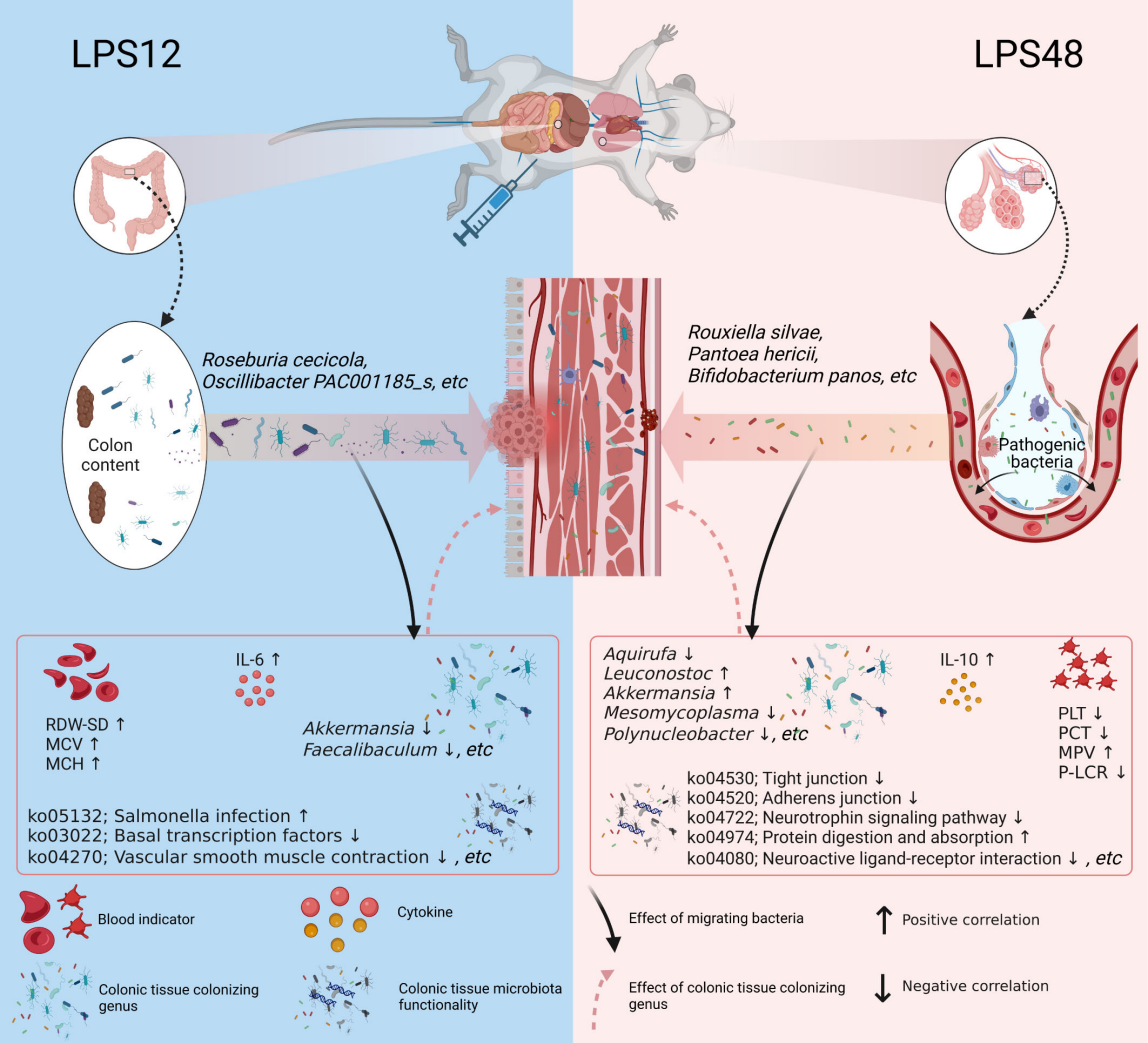
Schematic diagram depicting the dynamic changes of migratory microbial components in colon tissue at different time points during sepsis. After 12 hours of LPS injection, 57 characteristic microbial components of colon content translocated into colon tissue. Among them, *Roseburia cecicola* and *Oscillibacter* PAC001185_s were the predominant species among the translocated. 48 hours after LPS injection, 36 characteristic microbial components of lung tissue appeared in colon tissue. Among them, *Rouxiella silvae*, *Pantoea hericii*, and *Bifidobacterium panos* were the predominant species among the translocated. The migratory microbial components in colon tissue were significantly correlated with blood indicators as well as the colonizing genus abundance and microbiota functionality in colon tissue, suggesting that these migrating bacterial species may be directly or indirectly involved in the pathophysiological processes of sepsis by influencing the structure and function of the colonic microbiota. Created with BioRender.com.

The core finding of our study is that the microbiota of colon tissue changes dynamically with time after LPS injection, which involves the translocation of microbial components from colon content and lung tissue to colon tissue at different time points after LPS injection. Among them, multiple migrating microbial components were significantly associated with multiple blood indicators, colonizing genera, and microbiota functionalities of colon tissue (**Figure 6**). Our results suggest that these migrating microbial components may be involved in the systemic pathophysiologic response of sepsis either directly or indirectly by affecting the ecology of colon tissue microbiota.

The gastrointestinal tract is generally damaged during sepsis. Studies have shown that sepsis results in dysfunction and increased permeability of the intestinal barrier (Deitch, 2002; Fink, 2003; Yoseph et al., 2016; Wells et al., 2017). This theoretically prompts the possibility of bacterial translocation across habitats. Moreover, it can be speculated that the cross-habitat bacterial translocation can lead to further tissue damage by triggering inflammation in the host. In fact, several lines of evidence have shown that microbial components of the intestinal lumen contents can be translocated across the intestinal mucosa during sepsis and exacerbate the damage to the organism. O’Boyle et al. reported that complications of sepsis were significantly more prevalent in patients with bacterial translocation in surgical cases compared with patients without microorganisms found in the mesenteric lymph nodes at the time of dissection (O’Boyle et al., 1998; MacFie, 2004). Additional studies have also reported that bacterial translocation occurs in mouse models of fecal ligation and puncture-induced sepsis and that this is associated with higher mortality rates (Fredenburgh et al., 2011).

However, since the alteration of intestinal permeability and translocation of bacteria during the onset of sepsis is transient, the presence of bacteria in the blood or mesenteric lymph cannot be effectively detected by bacterial culture methods (Wiest and Rath, 2003; Dickson et al., 2016). Therefore, the dynamics of bacteria in colon tissues during the development of sepsis remain to be clarified. Sequencing-based approaches depend only on the presence of bacterial DNA and are much more sensitive than previous culture-based methods, so in this study, we utilized 16S rRNA to study the bacterial composition in colon tissue. Considering that sepsis has dynamic and temporally heterogeneous clinical and biological courses, we studied two time points, 12 and 48 h after LPS injection.

In the present study, by conducting microbial source tracking analysis, characteristic microbial component identification, and abundance difference analysis, we found that bacteria migrating to colon tissue at 12 h of LPS-induced sepsis were mainly derived from colon content, and bacteria migrating to colon tissue at 48 h of LPS-induced sepsis were mainly derived from lung tissue. According to Wang et al., an increase in intestinal permeability can occur as early as 4 hours after the onset of sepsis (Wang et al., 2001). Yoseph et al. also found that increased intestinal permeability can peak at 6–12 h during the onset of sepsis (Yoseph et al., 2016). Therefore, the presence of multiple bacteria originating from colon content in colon tissue in the early stage of sepsis (12 h after LPS injection) found in this study may be caused by the increased intestinal permeability and impaired intestinal mucosal barrier in the early stage of sepsis. Moreover, several papers have also reported that lung tissue damage can occur in the later stages of sepsis. Shanshan Cai et al. reported that lung injury, including hemorrhage, edema, alveolar wall thickening, and inflammatory cell infiltration, occurs 18–72 h after LPS injection in rats (Cai et al., 2009; Li et al., 2019). Similarly, Martínez-González et al. found that LPS-induced mice experienced marked pro-inflammatory alterations in the lungs, such as pulmonary vascular leakage, edema, and immune cell shedding, 48 h after endotoxin challenge (Martinez-Gonzalez et al., 2013). Furthermore, a recent study by Sze et al. suggested that bacteria are translocated from the lungs to the intestine through the blood within 24 h after acute lung injury (Sze et al., 2014). Based on these evidences, we speculate that the presence of multiple bacteria originating from the lung tissue in the colon tissue 48 hours after LPS injection found in this study may be associated with impaired respiratory membrane barrier function during the late stages of sepsis. In addition, when comparing the number of bacterial components that transiently migrated into the colon tissue at different time points, it is also interesting to observe that compared to 12h, there was a significant decrease (from 57 to 7) in the number of bacteria migrated from colon contents to colon tissue at 48h. This indicates that the bacteria that migrated to the colon tissue at 12h were unable to establish long-term colonization. We speculate this phenomenon may be related to the activation of local immunity in the colon tissues. That is, at 12h post LPS stimulation, intestinal mucosal injury occurs and allows a large number of colon content bacteria to migrate into the colon tissues. These bacteria are then gradually eliminated due to the activation of the local mucosal immune response. Taking together, the microbiome of colon tissues can change dynamically during the development of sepsis, where different stages of sepsis involve the translocation of bacteria from different parts of the organism.

In a taxonomic annotation of the microbial components of the colon content translocated into the colon tissue at 12 h after LPS injection, we found that 8 and 6 migrating ZOTUs belonged to *Roseburia cecicola* and *Oscillibacter PAC001185_s*, respectively, indicating that these two species were the main species translocated from the colon content to the colon tissue. Some studies have shown that the genera *Roseburia* and *Oscillibacter* are significantly associated with intestinal barrier damage in patients with sepsis. For example, Yang et al. found that elevated levels of intestinal *Roseburia* in patients with sepsis were significantly correlated with greater impairment of intestinal barrier function (Yang et al., 2021). Yan et al. found that an increase in the abundance of the genus *Oscillibacter* in mice led to an increase in intestinal permeability, and there was a negative correlation between *Oscillibacter* abundance in the colon and barrier function parameters (Lam et al., 2012).

In a correlation analysis between bacteria translocated 12 h after LPS injection and hematological parameters, we found a strong positive correlation between the abundance of zotu2880 (belonging to *Oscillibacter PAC001185_s*) in colon tissue and RBC-SD, a parameter related to a decrease in RBC deformability (Patel et al., 2013). In addition, the abundances of zotu1231 (belonging to *Prevotella hominis*) and zotu11711 (belonging to *Sporobacter PAC001257_s*) in colon tissue were strongly positively correlated with MPV and MCH, which are both related to a decrease in erythrocyte oxygen transport capacity (George-Gay and Parker, 2003). Many studies have reported that decreased RBC deformability and elevated RBC distribution width (RDW) are predictive factors of a worse prognosis of sepsis (Jo et al., 2013; Wang et al., 2018; Hu et al., 2020; Moreno-Torres et al., 2022). Our data suggest that the translocation of certain bacteria from the colon content that occurs in the colon tissue early in sepsis may cause alterations in the morphology of blood components, which may cause microcirculatory damage.

Moreover, some of these translocated bacteria were also associated with serum cytokine levels. We found a strong positive correlation between the abundance of zotu3155 (belonging to *Agathobaculum AY239438_s*) and the serum IL-6 level. It has been reported that the increase in serum IL-6 is an early marker for the diagnosis of neonatal sepsis (Ganesan et al., 2016; Shoukry et al., 2021). In the early stages of sepsis, a significant increase in IL-6 levels occurs as a surrogate marker of the pro-inflammatory response and is associated with increased intestinal permeability (Wang et al., 2001). These results suggest that the translocation of *Agathobaculum AY239438_s* to colon tissue in the early stages of sepsis may trigger local and systemic inflammatory responses and thus play a role in the elevated intestinal permeability.

Regarding the association of these microbial components that migrate early in sepsis with colon colonizing bacterial genera, we found that the abundance of zotu11152 (belonging to *PAC001296_g AB626952_s*) was strongly negatively correlated with the genus *Akkermansia*, and the abundance of zotu23 (belonging to *Helicobacter rodentium*) was strongly negatively correlated with the genus *Faecalibaculum*. The genus *Akkermansia* is a group of Gram-negative strictly anaerobic bacteria and has a role in maintaining the integrity of the mucosal barrier (Zhang et al., 2017; Peng et al., 2022). It was reported that an increase in the abundance of intestinal *Akkermansia* had a protective effect on the integrity of the intestinal mucosa (Huang et al., 2021), and oral administration of *Akkermansia* could significantly alleviate mucosal inflammation (Bian et al., 2019). Further studies in mouse models have shown that *Akkermansia* can reduce inflammation caused by metabolic endotoxemia by restoring the intestinal barrier (Wang et al., 2014; Fujisaka et al., 2020). The genus *Faecalibaculum* is a group of intestinal beneficial anaerobic bacteria that is considered one of the biomarkers of human health (Ferreira-Halder et al., 2017). It was reported that *Faecalibaculum* maintains the integrity of the colon wall by producing the energy needed for colon cells and also plays an important role in reducing inflammation by inhibiting the synthesis of pro-inflammatory cytokines such as IL-6 and IL-12 (Sokol et al., 2008; Sokol et al., 2009; Miquel et al., 2013). Based on these observations, we speculate that microbial components translocated from colon content into colon tissue early in sepsis may be indirectly involved in intestinal injury or exacerbation of sepsis symptoms by affecting colonizing bacterial genera in colon tissue.

In a taxonomic annotation of the microbial components of the lung tissue translocated into the colon tissue at 48 h after LPS injection, we found that *Rouxiella silvae*, *Pantoea hericii*, and *Bifidobacterium panos* were the predominant species translocated from the lung tissue to the colon tissue. *Rouxiella silvae* and *Pantoea hericii* are common bacteria in soil and water (Rong et al., 2016; Le Fleche-Mateos et al., 2017). A recent study on intestinal transplantation of *Rouxiella* bacteria found that it can regulate intestinal local immunity and intestinal motility (Yahfoufi et al., 2021). Other studies have shown that *Pantoea* bacteria can cause sepsis in infants when they enter the bloodstream (Mani and Nair, 2021). *Bifidobacterium panos* belongs to the genus *Bifidobacterium*. It is well known that *Bifidobacterium* is a common bacterial genus in the intestinal tract. However, in the analysis of the characteristic microbial components of the three habitats in this study, we found that *Bifidobacterium panos* was a characteristic bacterium of lung tissue. To resolve this confusion, we reviewed the literature and found only one report on *Bifidobacterium panos*, reporting it as a species first isolated from chimpanzee feces in the Czech Republic in 2020 (Neuzil-Bunesova et al., 2021), suggesting that this species is not a common bacterium in colon tissue. In fact, our results also demonstrated that the abundance of *Bifidobacterium panos* in both colon tissue and colon content was extremely low. There is no report on the physiological role of *Bifidobacterium panos* so far. At present, there are several reports discussing the translocation of intestinal bacteria to lung tissue during sepsis, but reports on the translocation of bacteria from lung tissue to intestinal tissue are very limited. To the best of our knowledge, the present study is the first to describe the specific species of lung tissue that migrate to colon tissue.

In the association analysis of the bacterial components that migrated from lung tissue to colon tissue 48 h after LPS injection with hematological parameters and serum cytokine levels, the abundance of 4 ZOTUs belonging to *Bifidobacterium panos* migrating to colon tissue was found to be significantly associated with 5 blood physiological indicators, including IL-10, PLT, PCT, MPV, and P-LCR. All of these blood physiological indicators were reported to be significantly associated with sepsis severity. For example, high levels of IL-10 have been shown to be a useful predictor of severity in septic shock and death and have been correlated with poor prognosis of sepsis in adults (Derkx et al., 1995; Gogos et al., 2000). Increased platelet volume and size reflects the existence of a thrombotic and inflammatory milieu, which has been used to predict poor clinical outcomes in patients with severe sepsis (Kim et al., 2015; Tajarernmuang et al., 2016; Lee et al., 2018). The development of thrombocytopenia during a septic episode is recognized as a significant event associated with multiple organ failure and increased mortality (Kaukonen et al., 2015). Moreover, we also found that the 3 ZOTUs belonging to *Bifidobacterium panos* were strongly correlated with multiple colonizing genera in colon tissue, such as *Leuconostoc*, *Akkermansia*, *Mesomycoplasma*, *Aquirufa*, and *Polynucleobacter*. Among them, the genera *Leuconostoc*, *Akkermansia*, and *Polynucleobacter* have been reported to play critical roles in the development of sepsis or bacteremia (Patel et al., 2012; Pavon-Delgado et al., 2015; Morowitz et al., 2017; Adelman et al., 2020; Wang et al., 2021). These results suggest that the migration of *Bifidobacterium panos* to colon tissue in late sepsis may be an important event in the progression of sepsis, which is worthy of further study.

We also analyzed the correlation between the microbial components migrating to the colon tissue in different stages of sepsis and the functionality of the colon microbiota. We found several significant correlations. Among those correlated microbiota functionalities, the pathways of ko04270 (vascular smooth muscle contraction), ko04722 (neurotrophic signaling pathway), ko05132 (*Salmonella* infection), ko03022 (basal transcription factors), ko04974 (protein digestion and absorption), ko04080 (neuroactive ligand–receptor interaction), ko04520 (adherens junction), ko04530 (tight junction), and ko04722 (neurotrophin signaling pathway) are involved in multiple functions such as the intestinal barrier, neurotrophin signaling, protein digestion and absorption, and vasoconstriction and participate in the regeneration and physiological function of intestinal tissues from different aspects (Arevalo and Wu, 2006; Akata, 2007; Zihni et al., 2016; Wemyss and Pearson, 2019). The discovery of these associations provides further insight into the biological significance of the migration of bacteria occurring at different stages of sepsis.

In summary, we analyzed the dynamic changes of microbiota and bacterial translocation in colon tissues at different stages of sepsis in this study, and found that translocation of bacteria from colon contents and lung tissue to colon tissue occurred during early and late sepsis. We obtained species-specific information about these migrating bacterial components and also found that they were broadly associated with blood indicators, colonizing genus abundance, and microbiota functionality in colon tissue, suggesting these migrating bacteria may be involved in the pathophysiological processes of sepsis either through direct action or indirectly by influencing the structure and function of colonic microbiota.

However, it is important to acknowledge certain limitations in our study. One limitation is the exclusive use of male rats, which was a decision based on avoiding the potential interference introduced by the estrous cycle in female rats (Lakbar et al., 2023). The study also had an observational nature without interventions to demonstrate the functional impact of microbial translocation. Future investigations with validation across genders, intervention studies, and mechanistic characterizations will help systematically elucidate the intricate roles of microbes during sepsis pathogenesis and translate the findings into clinical applications. Despite these limitations, as the first study providing species-specific information about these migrating bacteria and describing the temporal characteristics of bacterial translocation from different source sites, this work provides important insights into understanding the potential role of bacterial translocation in the pathogenesis of sepsis. Further studies on bacterial translocation between different habitats during sepsis could provide a more targeted approach to the prevention and treatment of sepsis.

## Data availability statement

The datasets generated and/or analyzed during the current study are available in the SRA database repository, Accession No: PRJNA1015001/PRJNA755451. The code scripts used for analysis and visualization have been deposited on GitHub (https://github.com/XuHaowk/my_16s).

## Ethics statement

The animal study was approved by The Ethics Committee of Minzu University of China. The study was conducted in accordance with the local legislation and institutional requirements.

## Author contributions

HX: Data curation, Visualization, Writing – original draft, Writing – review & editing. JY: Investigation, Resources, Writing – review & editing. WH: Writing – review & editing. LP: Writing – review & editing. YH: Writing – review & editing. XW: Writing – review & editing. ZT: Funding acquisition, Writing – review & editing. XZ: Funding acquisition, Writing – review & editing. EW: Writing – review & editing. YL: Resources, Writing – review & editing.

## Supplementary material

Supplementary Table 1 Detailed list of characteristic ZOTUs of different habitats and their exact sequences.

Supplementary Data Sheet 1 Chi-square test of different sources of characteristic microbial components in colon tissues at different time points after LPS stimulation.

Supplementary Data Sheet 2 The variation in ZOTU abundance in the different groups.
