# Supplementary material for: Dynamic changes in the migratory microbial components of colon tissue during different periods of sepsis in an LPS-induced rat model
Source: Front Cell Infect Microbiol. 2024 Jan 15;13:1330087. doi: 10.3389/fcimb.2023.1330087 (PMC10822926; doi:10.3389/fcimb.2023.1330087)
Supplement: Supplementary Data Sheet 1 — Chi-square test of different sources of characteristic microbial components in colon tissues at different time points after LPS stimulation. [file DataSheet_1.docx]

**Chi-square test of different sources of characteristic microbial components in colon tissues 12h after LPS stimulation**

**Single Table Analysis**

|  |  |  |  |  |
| --- | --- | --- | --- | --- |
|  | 57 | 1655 | 1712 | Characteristic microbial components of colon content of the control group |
|  | 4 | 668 | 672 | Characteristic microbial components of lung tissue of the control group |
|  | 61 | 2323 |  |  |
|  | Characteristic microbial components of colon tissue of the LPS12 group | Microbial components that were not characteristic of the colon tissue of the LPS12 group |  |  |

| **Chi Square and Exact Measures of Association** | | | |
| --- | --- | --- | --- |
| **Test** | **Value** | **p-value(1-tail)** | **p-value(2-tail)** |
| Mid-P exact |  | 0.00001217 | 0.00002434 |

All expected values (row total*column total/grand total) are >=5

OK to use chi square.

**Chi-square test of different sources of characteristic microbial components in colon tissues 48h after LPS stimulation**

**Single Table Analysis**

|  |  |  |  |  |
| --- | --- | --- | --- | --- |
|  | 7 | 1705 | 1712 | Characteristic microbial components of colon content of the control group |
|  | 36 | 636 | 672 | Characteristic microbial components of lung tissue of the control group |
|  | 43 | 2341 |  |  |
|  | Characteristic microbial components of colon tissue of the LPS48 group | Microbial components that were not characteristic of the colon tissue of the LPS48 group |  |  |

| **Chi Square and Exact Measures of Association** | | | |
| --- | --- | --- | --- |
| **Test** | **Value** | **p-value(1-tail)** | **p-value(2-tail)** |
| Mid-P exact |  | <0.0000001(P) | <0.0000001 |

All expected values (row total*column total/grand total) are >=5

OK to use chi square.
