# Supplementary material for: Dynamic changes in the migratory microbial components of colon tissue during different periods of sepsis in an LPS-induced rat model
Source: Front Cell Infect Microbiol. 2024 Jan 15;13:1330087. doi: 10.3389/fcimb.2023.1330087 (PMC10822926; doi:10.3389/fcimb.2023.1330087)
Supplement: Supplementary Data Sheet 2 — The variation in ZOTU abundance in the different groups. [file DataSheet_2.docx]

**Supplementary Figure S1**


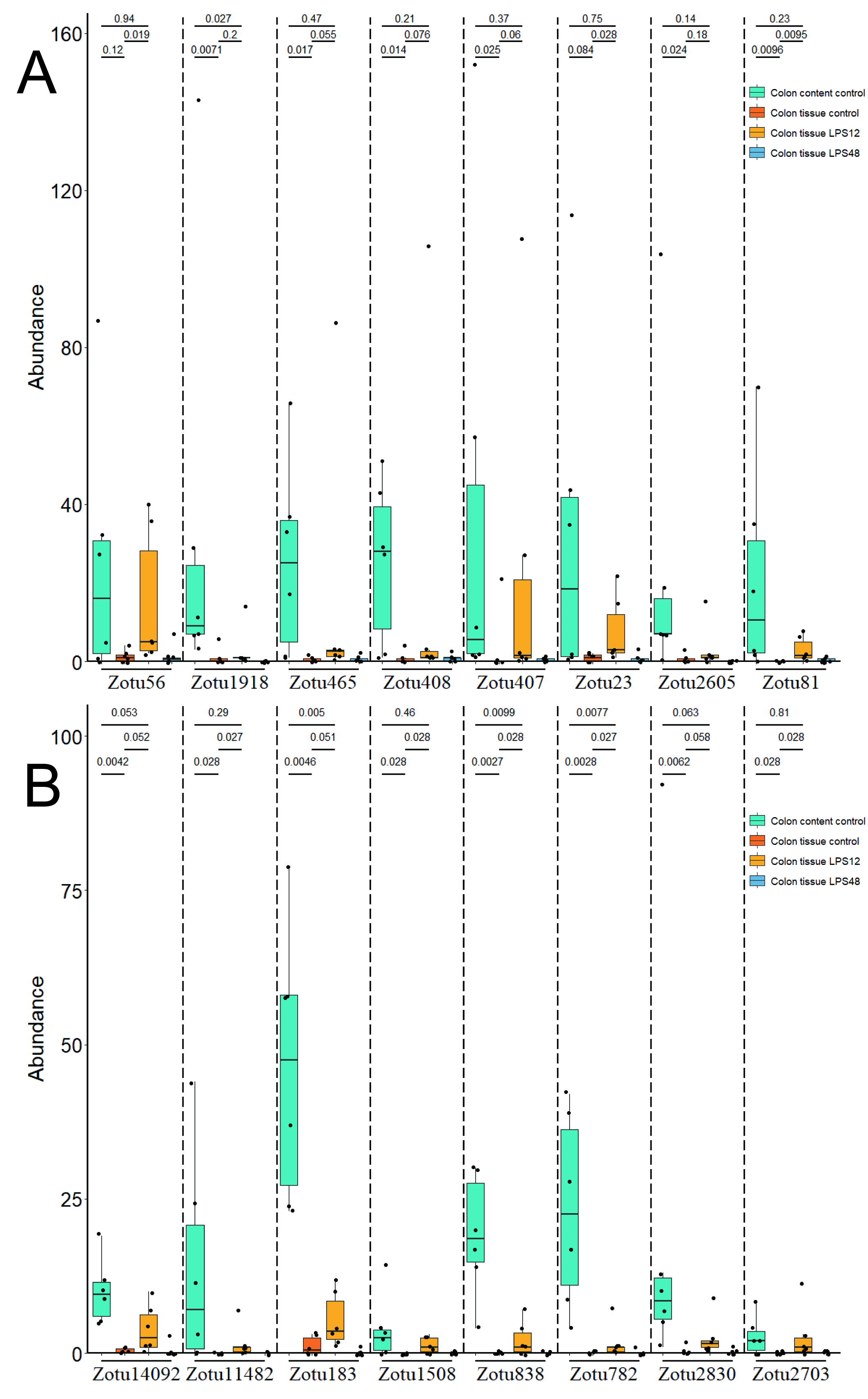


**Supplementary Figure S1**
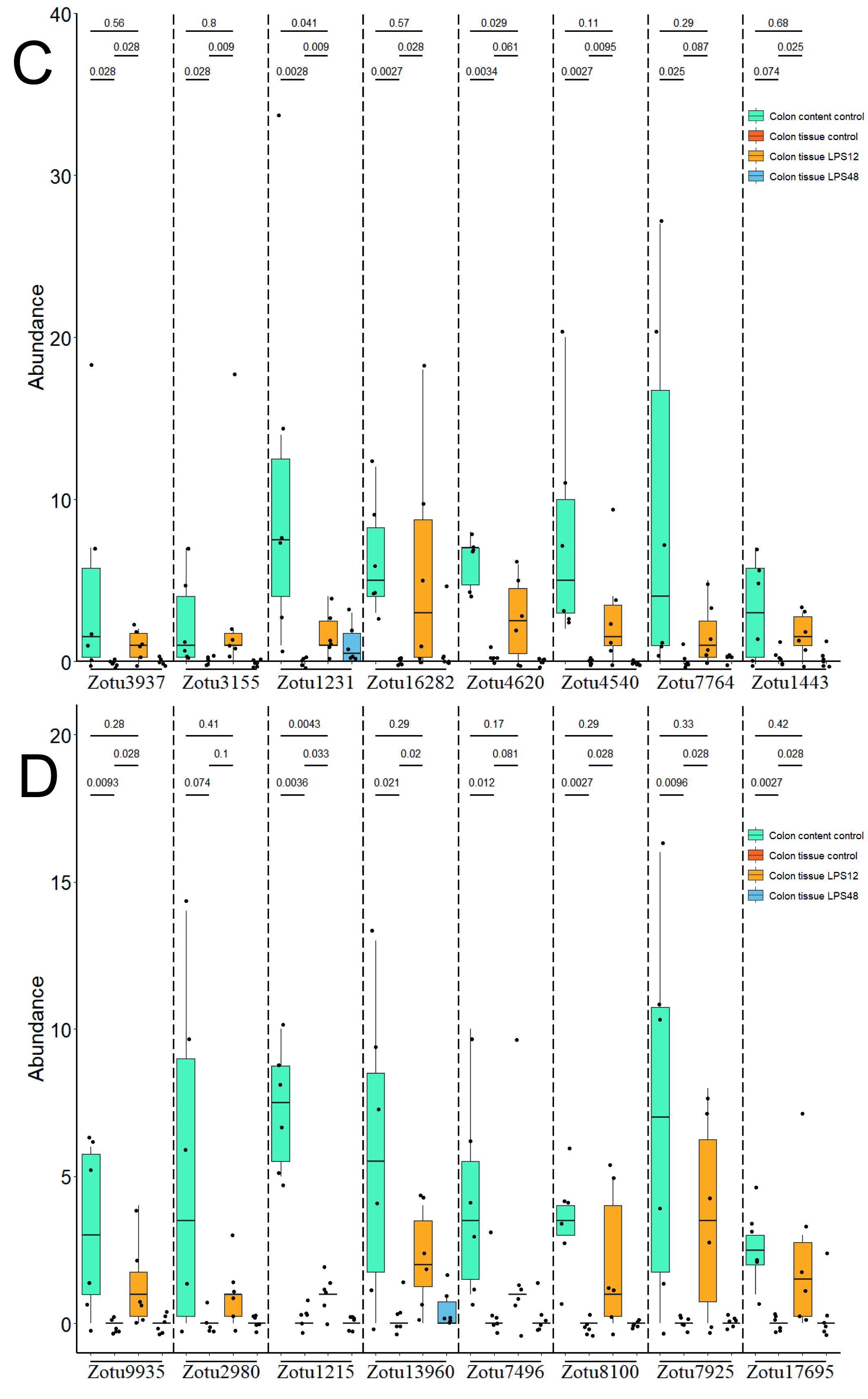
 **Supplementary Figure S1**


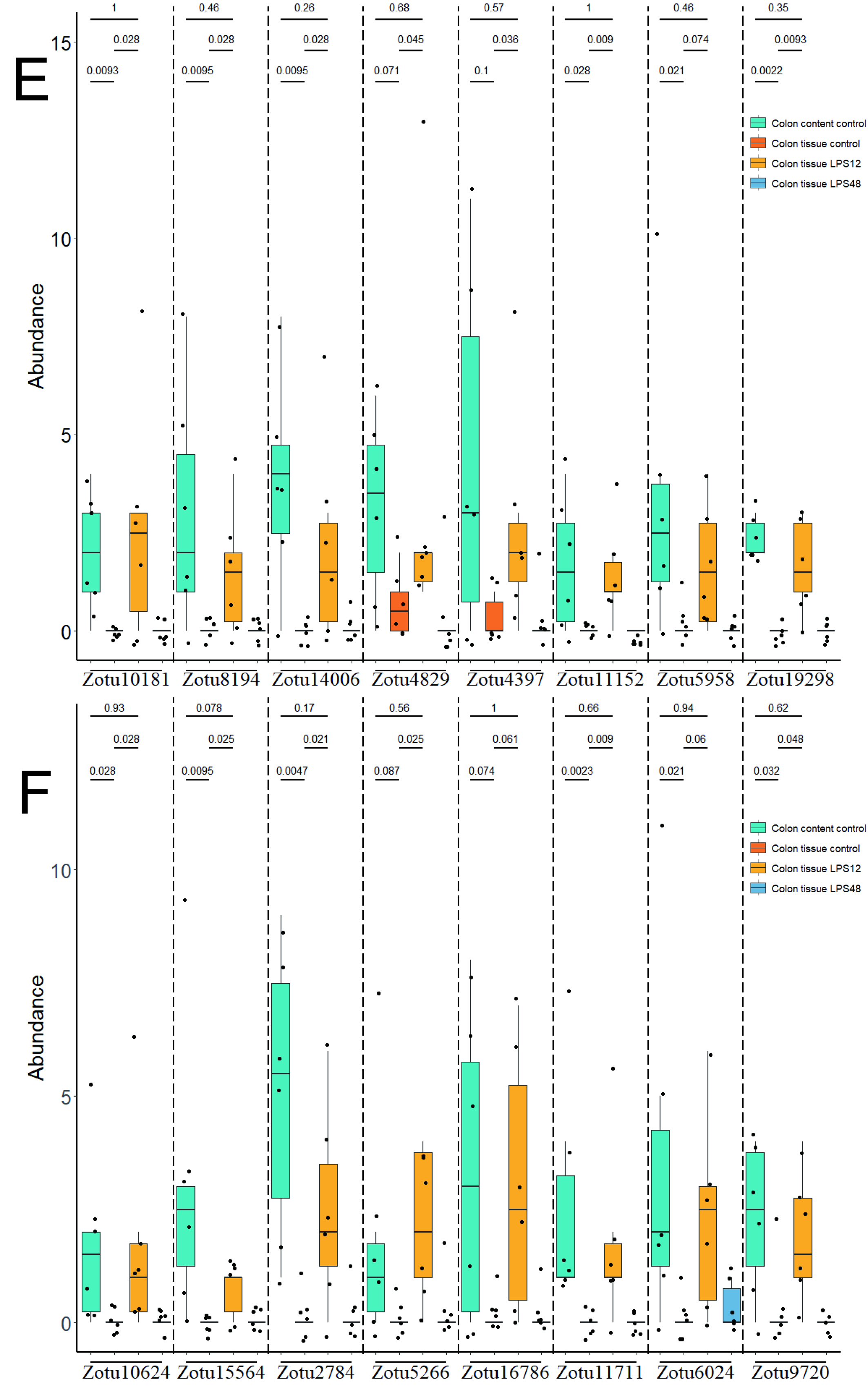
 **Supplementary Figure S1**
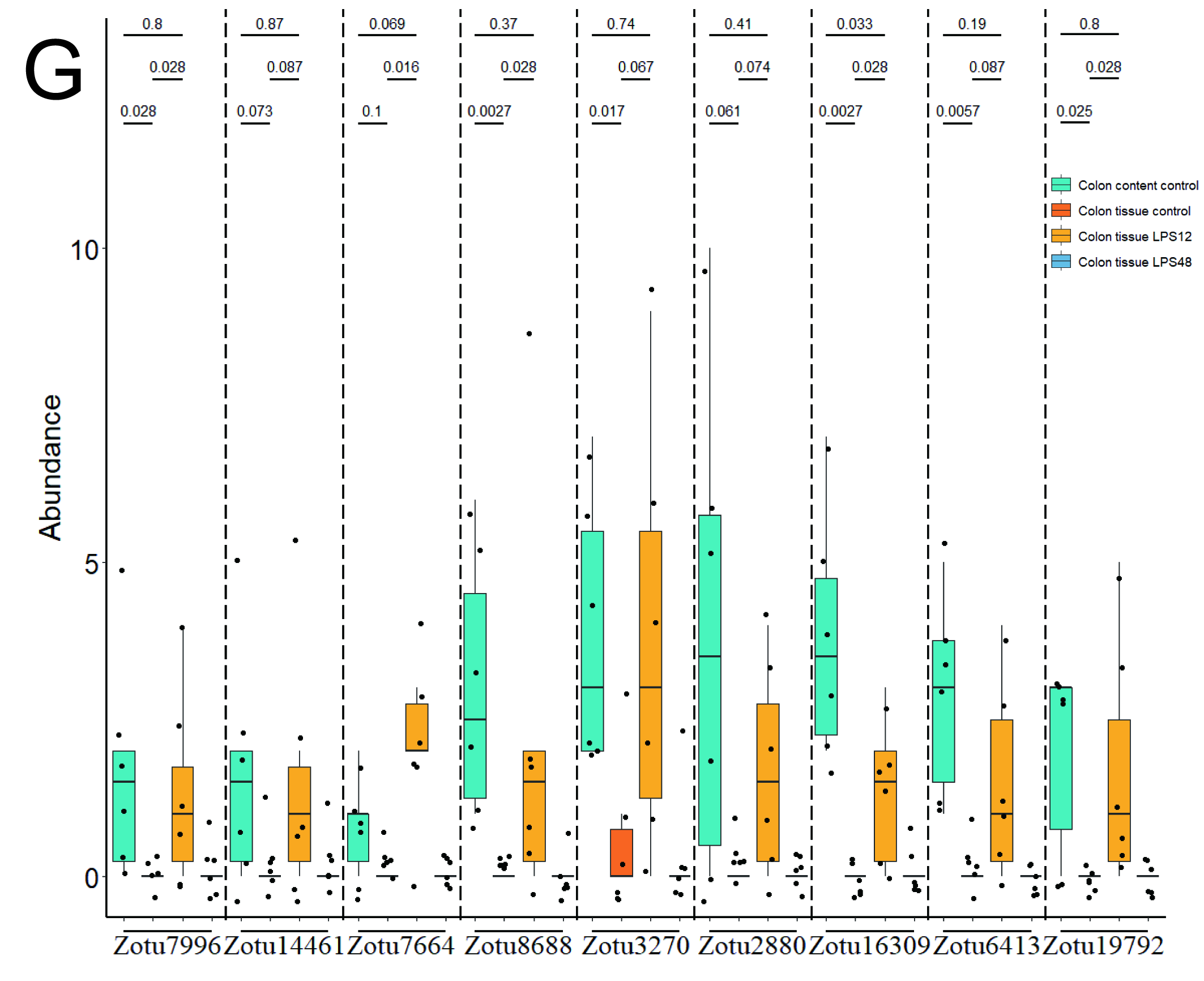


**Figure S1. Comparison of the abundance of 57 colon content source characteristic ZOTUs in the colon content control, colon tissue control and colon tissue LPS12 groups.**

**Supplementary Figure S2**

**
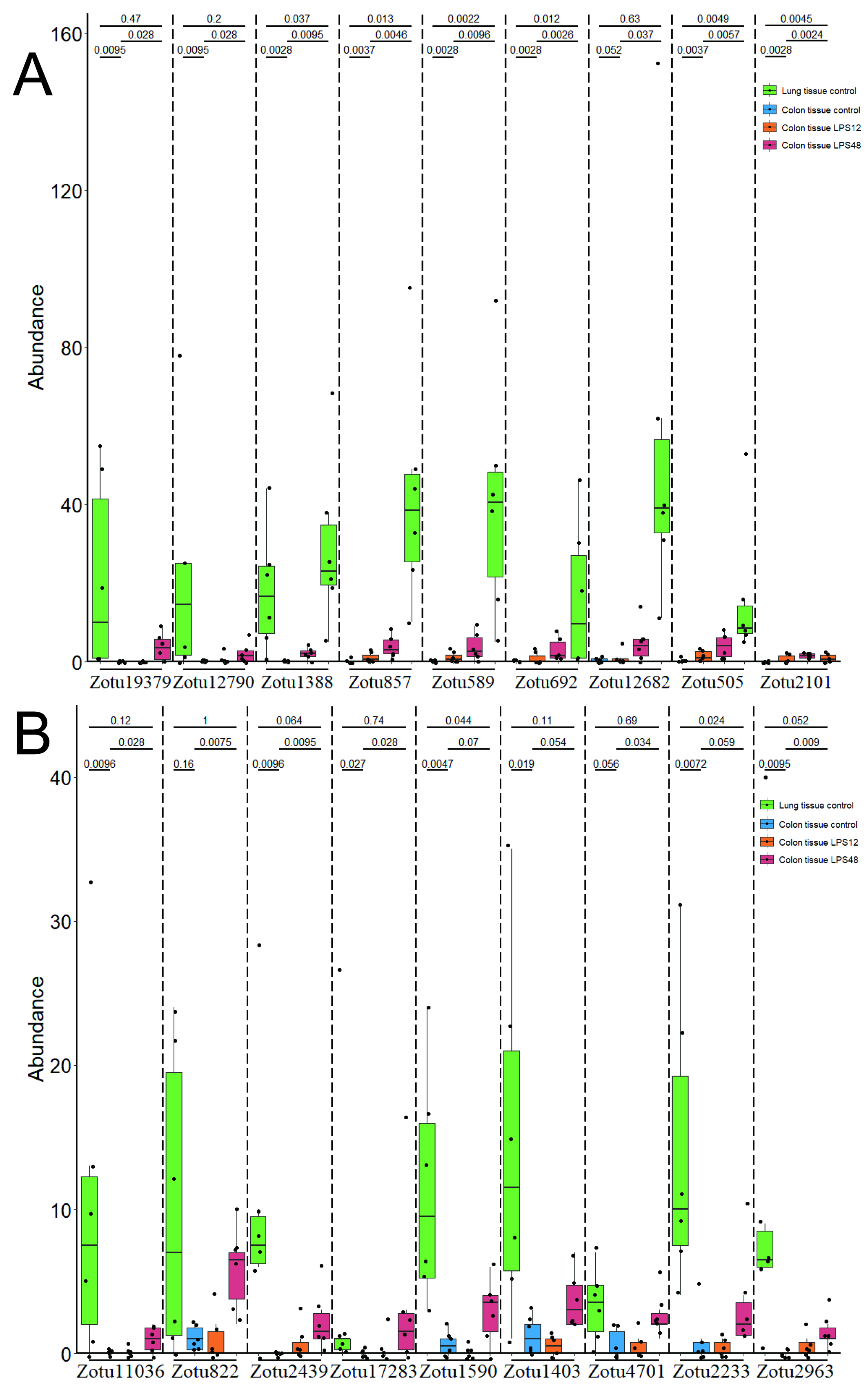
**

**Supplementary Figure S2**

**
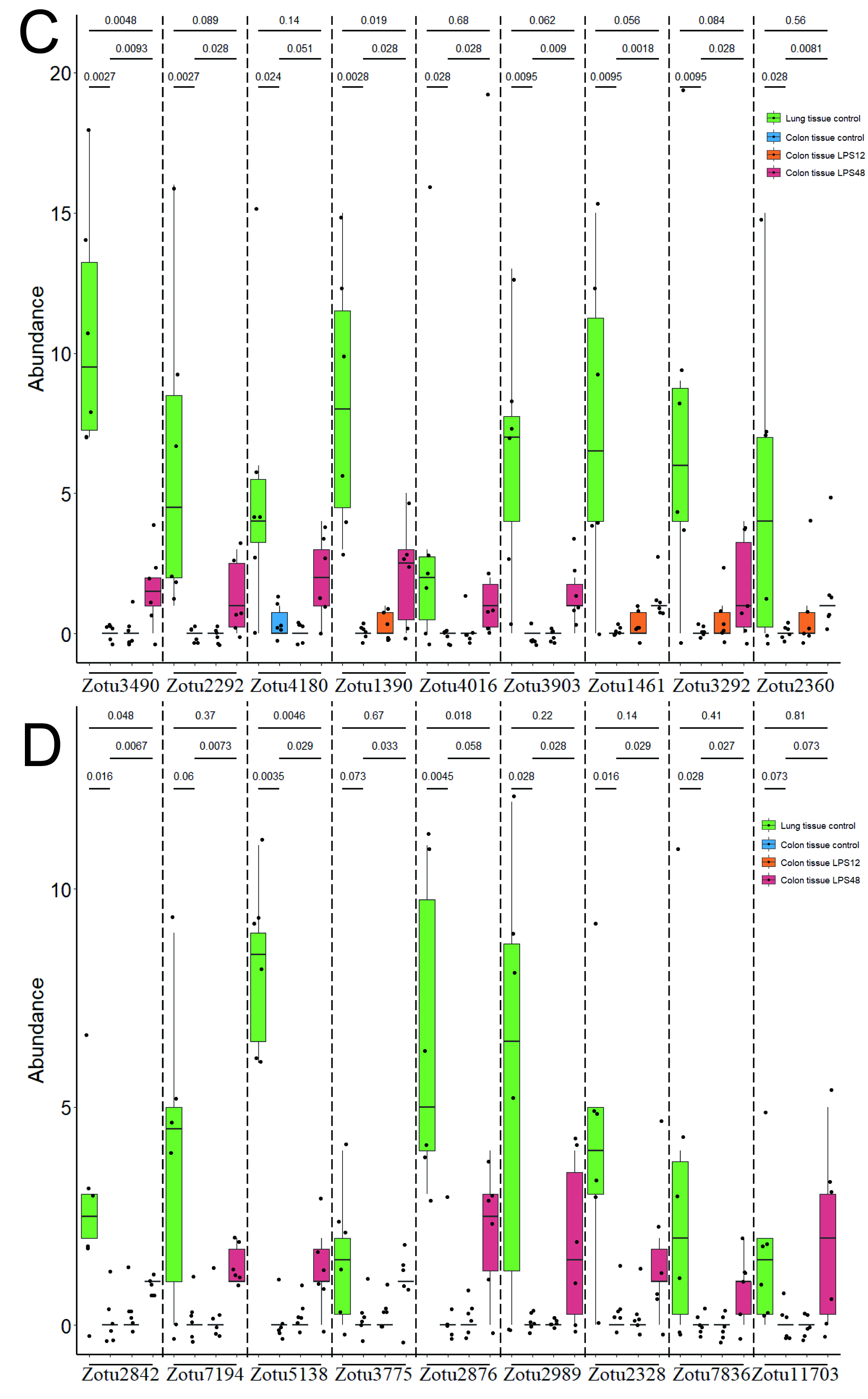
**

**Figure S2. Comparison of the abundance of 36 lung tissue source characteristic ZOTUs in the lung tissue control, colon tissue control and colon tissue LPS48 groups.**
